# Anti-RBD Antibody Levels and IFN-γ-Specific T Cell Response Are Associated with a More Rapid Swab Reversion in Patients with Multiple Sclerosis after the Booster Dose of COVID-19 Vaccination

**DOI:** 10.3390/vaccines12080926

**Published:** 2024-08-19

**Authors:** Alessandra Aiello, Serena Ruggieri, Assunta Navarra, Carla Tortorella, Valentina Vanini, Shalom Haggiag, Luca Prosperini, Gilda Cuzzi, Andrea Salmi, Maria Esmeralda Quartuccio, Anna Maria Gerarda Altera, Silvia Meschi, Giulia Matusali, Serena Vita, Simonetta Galgani, Fabrizio Maggi, Emanuele Nicastri, Claudio Gasperini, Delia Goletti

**Affiliations:** 1 Translational Research Unit, National Institute for Infectious Diseases Lazzaro Spallanzani-Istituto di Ricovero e Cura a Carattere Scientifico (IRCCS), 00149 Rome, Italy; alessandra.aiello@inmi.it (A.A.); valentina.vanini@inmi.it (V.V.); gilda.cuzzi@inmi.it (G.C.); andrea.salmi@inmi.it (A.S.); annamaria.altera@inmi.it (A.M.G.A.); 2 Department of Neurosciences, San Camillo Forlanini Hospital, 00152 Rome, Italy; serena.ruggieri@gmail.com (S.R.); carla.tortorella@gmail.com (C.T.); shaggiag@scamilloforlanini.rm.it (S.H.); quartuccioe@libero.it (M.E.Q.); galgasi@tiscali.it (S.G.); cgasperini@scamilloforlanini.rm.it (C.G.); 3 Clinical Epidemiology Unit, National Institute for Infectious Disease Lazzaro Spallanzani-Istituto di Ricovero e Cura a Carattere Scientifico (IRCCS), 00149 Rome, Italy; assunta.navarra@inmi.it; 4 Simple Operating Unit Technical Healthcare Professions , National Institute for Infectious Diseases Lazzaro Spallanzani-Istituto di Ricovero e Cura a Carattere Scientifico (IRCCS), 00149 Rome, Italy; 5 Laboratory of Virology, National Institute for Infectious Diseases Lazzaro Spallanzani-Istituto di Ricovero e Cura a Carattere Scientifico (IRCCS), 00149 Rome, Italy; silvia.meschi@inmi.it (S.M.); giulia.matusali@inmi.it (G.M.); fabrizio.maggi@inmi.it (F.M.); 6 Clinical Division of Infectious Diseases, National Institute for Infectious Diseases Lazzaro Spallanzani-Istituto di Ricovero e Cura a Carattere Scientifico (IRCCS), 00149 Rome, Italy; serena.vita@inmi.it (S.V.); emanuele.nicastri@inmi.it (E.N.)

**Keywords:** COVID-19 vaccination, SARS-CoV-2, multiple sclerosis, disease-modifying therapies, antibody response, T cell response, breakthrough infection

## Abstract

This study investigated the incidence and severity of SARS-CoV-2 breakthrough infections (BIs) and the time to swab reversion in patients with multiple sclerosis (PwMS) after the booster dose of COVID-19 mRNA vaccines. We enrolled 64 PwMS who had completed the three-dose mRNA vaccine schedule and had never experienced COVID-19 before. Among the 64 PwMS, 43.8% had BIs with a median time since the third vaccine dose of 155 days. BIs occurred more frequently in ocrelizumab-treated patients (64.7%). Patients with a relapsing-remitting MS course showed a reduced incidence of BIs compared with those with a primary-progressive disease (*p* = 0.002). Having anti-receptor-binding domain (RBD) antibodies represented a protective factor reducing the incidence of BIs by 60% (*p* = 0.042). The majority of BIs were mild, and the only severe COVID-19 cases were reported in patients with a high Expanded Disability Status Scale score (EDSS > 6). The median time for a negative swab was 11 days. Notably, fingolimod-treated patients take longer for a swab-negativization (*p* = 0.002). Conversely, having anti-RBD antibodies ≥ 809 BAU/mL and an IFN-γ-specific T cell response ≥ 16 pg/mL were associated with a shorter time to swab-negativization (*p* = 0.051 and *p* = 0.018, respectively). In conclusion, the immunological protection from SARS-CoV-2 infection may differ among PwMS according to DMTs.

## 1. Introduction

Coronavirus Disease-2019 (COVID-19) is a human-to-human transmissible disease caused by the Severe Acute Respiratory Syndrome Coronavirus 2 (SARS-CoV-2), that emerged in late 2019 as an outbreak and was subsequently declared a global health emergency by the World Health Organization (WHO) on 30 January 2020 [1].

The large-scale vaccination campaign starting at the beginning of 2021 has proven to be an effective measure to counteract the COVID-19 pandemic by limiting the spread of SARS-CoV-2 infections and reducing the risk of a severe disease outcome and hospitalization rate. This led the WHO to declare the end of the COVID-19 pandemic in May 2023. Nevertheless, COVID-19 still represents a threat to the public health of vulnerable categories [2,3,4], including patients with multiple sclerosis (PwMS) [5,6,7]. Besides COVID-19, other infectious diseases may have a more severe course in MS patients [8], and the COVID-19 clinical and scientific experience may represent a kind of “model” for the management of other infections as well.

Multiple sclerosis (MS) is a neurodegenerative and autoimmune disease that affects the central nervous system and represents the principal non-traumatic cause of disability among young adults in Western countries [9]. The majority of PwMS undergoes immunomodulatory or immunosuppressive disease-modifying therapies (DMTs), including ocrelizumab, fingolimod, cladribine, and interferon (IFN)-β [9]. DMTs administered in PwMS have a wide range of actions targeting the immune system and result in the potential impairment of humoral and/or cell-mediated immune responses to both infections and vaccines. For this reason, during the COVID-19 pandemic, the clinic management of PwMS was challenging and raised great concern.

Different studies reported that PwMS are more prone to contracting infections and present a higher risk of infection-related hospitalization and infection-related mortality [10,11,12,13,14].

Our and other groups showed that the majority of PwMS can mount humoral and/or T cell-specific immune responses to COVID-19 mRNA vaccines, albeit with a lower magnitude than healthy subjects [5,6,7,15,16,17]. To note, the magnitude of the immune response was different according to the ongoing DMTs. Particularly, fingolimod- and ocrelizumab-treated patients were those who presented a more compromised response to COVID-19 vaccination [5,6,7,16,18,19,20].

Despite the effectiveness of the vaccination campaign, breakthrough infections (BIs) continued to be reported owing to the emergence of SARS-CoV-2 variants escaping the host immune defense, concomitantly with the reduction of distancing measures and the decline of vaccine immunity over time [5,21,22,23,24,25]. The onset of new variants is likely favored by the ongoing replication of the virus, particularly in immunocompromised individuals who are more prone to establishing persistent infections [26].

It has been reported that the administration of the third dose of COVID-19 vaccines also contributes to protection against Omicron variants, by reducing the incidence and severity of BIs in the general population [27,28,29,30,31]. Regarding PwMS, studies reported in the literature have mainly focused on B-depleted patients [32,33,34], or have assessed the impact of clinical factors and/or antibody response on the risk of BIs without taking into account the T cell response [35,36,37,38,39,40]. Moreover, they do not investigate factors potentially associated with the time to swab reversion.

This study aimed to investigate the demographic, clinical, and immunological factors, both antibody and T cell responses, potentially associated with the risk of BIs, severity of the outcome, and time of swab negativization in the MS population treated with different DMTs after the third dose of COVID-19 mRNA vaccines. The study period overlapped with the onset of the Omicron variant, which was the predominant variant in Italy starting in January 2022 [41].

## 2. Materials and Methods

### 2.1. Study Cohort and Design

This is a prospective longitudinal single-center study. Patients diagnosed with MS according to the 2017 McDonald criteria [42] were enrolled at the MS Centre of the Department of Neurosciences of San Camillo Forlanini Hospital (Rome, Italy). We enrolled PwMS who were on treatment with ocrelizumab, fingolimod, cladribine, or IFN-β for at least 6 months from the enrolment and had received the three doses of COVID-19 mRNA vaccines (BNT162b2 or mRNA-1273). The exclusion criteria were as follows: previous SARS-CoV-2 infection based on a positive antigenic and/or molecular test by real-time polymerase chain reaction (PCR) on the swab sample and/or positive anti-nucleoprotein immunoglobulin G (anti-N-IgG) before the third vaccine dose, HIV infection, and age < 18 years.

**Figure 1:**
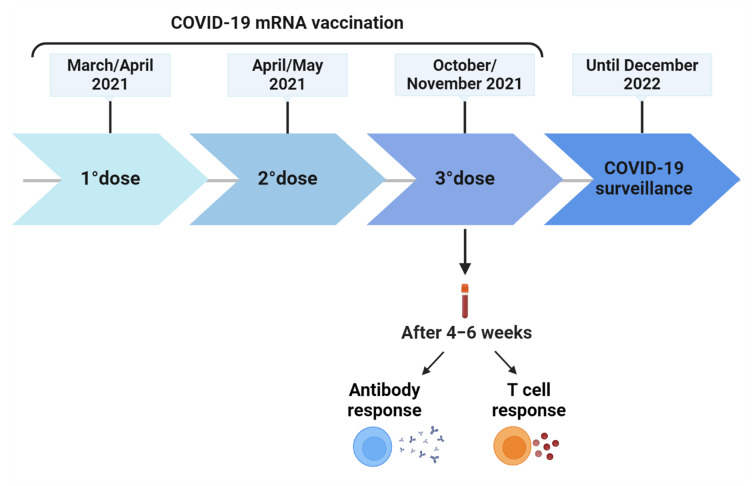
Timeline of COVID-19 vaccination schedule and surveillance. After 4–6 weeks from the third vaccine dose, blood samples were collected to evaluate antibody and T cell response. Abbreviations: COVID-19, Corona Virus Disease 2019. Created with BioRender.com.

The enrolment was initiated in March 2021, and the follow-up ended in December 2022. From a cohort previously evaluated in terms of antibody and T cell response to COVID-19 vaccination at baseline (2–4 weeks after the second dose) [6], after 24 weeks from the first dose, and 4–6 weeks after the third dose [5,7], a subgroup of 64 patients were followed up until a positive SARS-CoV-2 test or the administration of the fourth vaccine dose to evaluate the incidence rate of BIs (Figure 1). BIs were stratified based on their severity as mild, moderate, or severe [43]. Briefly, mild COVID-19 patients had any of the different signs and symptoms (i.e., fever, cough, sore throat, malaise, headache, etc.) but without shortness of breath, dyspnea, or abnormal chest imaging; moderate COVID-19 patients had lower respiratory disease and SpO2 ≥ 94% on room air, while severe COVID-19 patients required hospitalization.

Demographic and clinical data were collected at the time of the third dose, while the immunological data refer to the evaluation performed 4–6 weeks after the third dose. The laboratory procedures were carried out following the standardized protocol previously described [44].

This study was approved by the Ethical Committee of “L. Spallanzani” National Institute of Infectious Diseases (INMI)-IRCCS (approval numbers 319/2021 and 443/2021) and was conducted in agreement with the Declaration of Helsinki. A written informed consent was signed by all participants before taking part in the study.

### 2.2. Anti-SARS-CoV-2 Antibodies

The antibody response to COVID-19 vaccination was evaluated by assessing anti-receptor-binding domain (RBD) antibodies (Abs), which were expressed as binding antibody units (BAU)/mL, and whose cut-off to define a positive response was set at ≥ 7.1.

Concurrently, neutralizing antibodies were evaluated with a micro-neutralization assay (MNA) using the SARS-CoV-2/Human/ITA/PAVIA10734/2020 (provided by Fausto Baldanti, Pavia, Italy), as previously described [45]. The neutralization titer was expressed as the reciprocal of the highest serum dilution (MNA_90_) at which we observed at least 90% inhibition of the cytopathic effect. The neutralizing titer was considered positive if ≥ 10, corresponding to the first dilution tested.

The anti-nucleoprotein immunoglobulin G (Anti-N-IgG) was evaluated to screen the enrolled cohort for a previous SARS-CoV-2 infection. Subjects scored positive if index values [(sample (S)/Cutoff (CO)] ≥1.4. Both anti-RBD-IgG and anti-N-IgG were assessed following the manufacturer’s instructions (Architect^®^ i2000sr Abbott Diagnostics, Chicago, IL, USA).

### 2.3. IFN-γ-Spike-Specific T cell Response

The IFN-γ-spike specific T cell response was tested using a whole blood assay. The blood was collected in lithium heparinized tubes (BD Vacutainer, Becton Dickinson, Florence, Italy, Cat. 367526) and stimulated in a 48-well plate with a mixture of spike peptides derived from the SARS-CoV-2 Wuhan spike protein (PepTivator^®^Prot_S1, Prot_S, and Prot_S+, Miltenyi Biotec, Bergisch Gladbach, Germany, Cat. 130–127–048, Cat. 130–126–701 and Cat. 130–127–312, respectively) at a final concentration of 0.1 µg/mL [44]. The staphylococcal enterotoxin B (SEB) (Merck Life Science, Milan, Italy, Cat. S4881) was used as a positive control (Supplementary Figure S1). After an overnight incubation at 37 °C with 5% CO_2_, plasma was harvested and stored at −80 °C until use. The ELLA Simple Plex Human IFN-γ (3rd Gen.) Assay (Bio-Techne, Minneapolis, MN, USA, Cat. SPCKB-PS-002574) was used to quantify IFN-γ levels that defined a positive T cell response if ≥ 16 pg/mL. The values reported are subtracted from the unstimulated control value.

### 2.4. Statistical Analysis

The statistical analyses were performed using Stata (StataCorp. 2021. Stata Statistical Software: Release 17. College Station, TX, USA: StataCorp LLC.), R Project Software (version 4.2.1), and GraphPad (version 9.3.1) (GraphPad Prism, San Diego, CA, USA). Categorical variables were expressed as absolute and relative percentages, whereas the quantitative ones were reported as medians and interquartile ranges (IQR). The following statistical tests were used: the Kruskal–Wallis test to compare groups, the Mann–Whitney test for pairwise comparison, and the Fisher Exact test to compare categorical variables.

Univariable Poisson regressions were performed to estimate the incidence rate ratio (IRR) of SARS-CoV-2 infections according to the demographic, clinical, and immunological data. Variables with *p* < 0.2 were entered into the multivariable model, which was chosen with the minor Bayesian information criterion (BIC). The quantile regression analysis on the median was performed to estimate the time to swab conversion according to demographic, clinical, and immunological responses. Two-tailed *p*-values < 0.05 were considered statistically significant. Post-hoc power analysis has been performed for the main outcomes and for univariable models, to evaluate the statistical power of our sample size using G*Power software (version 3.1.9.7) [46]. According to the post hoc sample size calculation, our study was able to detect significant differences of IRRs of at least 2.5 for the primary outcome, incidence of SARS-CoV-2 breakthrough infections, and a reduction of about 75% in the analysis of antibody and T cell-specific immune response, after sample collection, with a power of 80% and an alpha error of 0.05.

## 3. Results

### 3.1. Characteristics of the Studied Population

Within a cohort of 134 PwMS previously evaluated for the immune response to COVID-19 vaccination [5,6,7], 64 individuals were followed up to investigate the BI incidence in the MS cohort. The demographic and clinical characteristics of the enrolled subjects are reported in Table 1. The cohort had a median time since MS diagnosis of 15 years and was characterized by a female predominance of 67%, as expected in autoimmune diseases. The majority of PwMS (92.2%) showed a relapsing-remitting phenotype. All the enrolled PwMs were under DMTs: 17 (26.6%) subjects were treated with ocrelizumab, 26 (40.6%) with fingolimod, 6 (9.4%) with cladribine, and 15 (23.4%) with IFN-β. A minority of subjects (21.9%) reported having comorbidities such as metabolic or cardiovascular diseases.

### 3.2. Incidence of Breakthrough Infections and Clinical Features

During the study period, out of 64 PwMS, 28 (43.8%) experienced BIs with a median time since the third vaccine dose of 155 days (Table 1). BIs occurred more frequently among PwMS treated with ocrelizumab (11/17, 64.7%) compared to those under fingolimod (10/26, 38.5%), IFN-β (6/15, 40%), and cladribine (1/6, 16.7%). Indeed, 39.3% of the total BIs were reported in subjects treated with ocrelizumab, 35.7% with fingolimod, 21.4% with IFN-β, and 3.6% with cladribine (Table 2). A different incidence of BIs was also observed according to sex; in the male population, 61.9% of subjects had a SARS-CoV-2 infection compared with only 35% of females. A significantly lower BI incidence rate was observed in PwMS with higher MS severity, reported as Expanded Disability Status Scale score (EDSS ≥ 3 IRR: 0.22, 95%CI: 0.08–0.66, *p* = 0.006). Moreover, PwMS with a relapsing-remitting disease showed an 88% lower rate of BI incidence (IRR: 0.12, 95%CI: 0.03–0.46, *p* = 0.002) compared to those with a primary-progressive disease (Table 2).

### 3.3. Incidence of Breakthrough Infections and Immune Response

**Figure 2:**
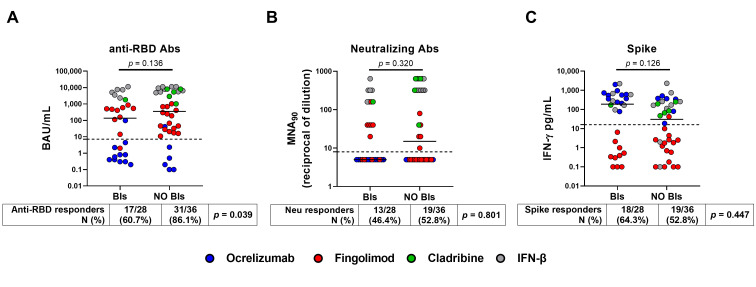
Antibody and IFN-γ-specific T cell responses after the third dose of COVID-19 vaccine. The enrolled PwMS (n = 64) were stratified into two groups: BIs group (n = 28), which included those who had COVID-19 after the third dose, and no BIs group (n = 36), which included those who did not have COVID-19 during the follow-up period. (**A**) Anti-RBD-IgG were expressed as binding antibody units (BAU)/mL, (**B**) neutralizing antibodies as the reciprocal of the dilution (MNA90), while (**C**) IFN-γ levels were subtracted from the unstimulated-control value and reported in pg/mL. The cut-off of each test is indicated by the black dashed line (anti-RBD-IgG: 7.1 BAU/mL; MNA90: 10 reciprocal dilution and spike: 16 pg/mL). Each colour dot represents a different treatment as shown in the legend. A Mann–Whitney test was performed for pairwise comparison and Fisher Exact test was to compare the proportion of responders. A *p*-value < 0.05 was considered significant. Abbreviations: COVID-19, Coronavirus Disease 2019; PwMS, patients with multiple sclerosis; BIs, breakthrough infections; RBD, receptor-binding domain; IgG, immunoglobulin; Abs, antibodies; IFN, interferon; N, number.

PwMS who did not have BIs showed a significantly higher seroconversion rate (31/36, 86.1%) after the third vaccine dose than those who had BIs (17/28, 60.7%) (*p* = 0.039). However, there were no significant differences in the magnitude of the antibody response after the third vaccine dose between PwMS who had BIs and those uninfected (135 BAU/mL, (IQR: 1–1337) vs. 352 BAU/mL, (IQR: 24–5513), *p* = 0.136), albeit a slightly higher antibody production was observed in those uninfected (Figure 2A). Among patients without BIs, fingolimod-treated patients were the most represented group with a frequency of 44% compared to 36% in patients with BIs (Table 2), and this likely influenced the overall result. Indeed, it is well known that fingolimod limits the ability for both T and B cell-specific responses. Therefore, although most patients receiving fingolimod therapy seroconverted, the antibody levels are low. Consequently, when comparing those without BIs with those who had BIs, even though there was a significant difference in the number of seroconverted individuals, there was no significant change in the antibody production between the two groups. Moreover, neither the neutralizing titer nor the IFN-γ T cell response significantly differed between PwMS who experienced BIs and the uninfected patients, both in terms of qualitative (*p* = 0.320 and *p* = 0.126, respectively) or quantitative response (*p* = 0.801 and *p* = 0.447, respectively) (Figure 2B,C).

In the univariable Poisson regression, we showed that the presence of an antibody response (anti-RBD ≥ 7.1 BAU/mL) represented a protective factor, reducing the incidence rate of BIs in the MS population by 67% (IRR: 0.33, 95%CI: 0.15–0.7, *p* = 0.004) (Table 3). After adjusting for EDSS score and disease phenotype, having anti-RBD response ≥ 7.1 BAU/mL still conferred a 60% reduction in the BI incidence rate (IRR: 0.40, 95%CI: 0.17–0.97, *p* = 0.042). Instead, no significant associations were found between the incidence rate of BIs and IFN-γ levels or neutralizing antibodies (*p* = 0.187 and *p* = 0.362, respectively).

### 3.4. COVID-19 Severity

The majority of BIs (21/28, 75%) were mild, while 17.9% (5/28) were moderate. Only two cases of severe COVID-19 requiring hospitalization were reported and involved male subjects under fingolimod and ocrelizumab treatment (Table 4).

Most PwMS who had mild COVID-19 were under fingolimod (8/21, 38.1%) therapy; differently, the majority of those with moderate COVID-19 were under ocrelizumab treatment (4/5, 80%). Regarding COVID-19 therapy, 11 PwMS with mild disease reported having assumed antiviral (n = 4) or monoclonal antibodies (n = 7), whereas 10 PwMS took non-steroidal anti-inflammatory drugs (NSAIDs) or paracetamol. Most patients with moderate COVID-19 were treated with monoclonal antibodies (Table 4). Stratifying by DMTs, we observed that monoclonal antibodies or antivirals were administered mostly to fingolimod- or ocrelizumab-treated patients, whereas patients undergoing IFN-β treatment took NSAIDs or paracetamol (Supplementary Table S1).

The two severe COVID-19 patients showed a higher EDSS score (median: 6.5, IQR: 6.5–6.5) compared to those with mild (median: 2.0, IQR: 0–2.5) or moderate disease (median: 1.5, IQR: 1.0–1.5) (Table 4).

None of the demographic, clinical, or immunological variables were significantly associated with COVID-19 severity.

### 3.5. Time of Swab Negativization

Among the 25 infected PwMS for whom the time of swab negativization was available, the median time to have a negative swab was 11 days (IQR: 9 –14 days) (Table 1). Despite antiviral or monoclonal antibody therapies, patients treated with fingolimod took a significantly longer time for a swab reversion (7 days, 95%CI: 2.84–11.16, *p* = 0.002) than PwMS under IFN-β (Table 5). A similar trend was observed also for ocrelizumab-treated patients, albeit not statistically significant (3 days, 95%CI: −1.16–7.16, *p* = 0.148). Instead, PwMS under cladribine and IFN-β showed similar times for swab reversion.

Conversely, having a high anti-RBD antibody titer (≥ 809 BAU/mL) and an IFN-γ-specific T cell response (≥ 16 pg/mL) were associated with a significantly shorter time for a negative swab (−6.00 days, 95%CI: −10.7–−1.3, *p* = 0.015; −5.00 days, 95%CI: −9.77–−0.23, *p* = 0.041, respectively). This result was also confirmed after adjusting for time elapsed from sample collection (anti-RBD: −4.39 days, 95%CI: −8.8–0.01, *p* = 0.051; T cell: −5.88 days, 95%CI: −10.65–−1.10, *p* = 0.018, respectively) (Table 5). Instead, neutralizing titers did not show any impact on the time needed for SARS-CoV-2 clearance.

## 4. Discussion

To the best of our knowledge, this is the first longitudinal prospective study investigating the demographic, clinical, and immunological factors potentially associated with both the risk of SARS-CoV-2 infection, disease severity, and the time for swab reversion in a cohort of PwMS treated with different DMTs after the third dose of COVID-19 vaccines.

The majority of the current studies have mainly focused on B-depleted patients [32,33,34] and the role played by clinical features and/or the only antibody response on the incidence and severity of BIs [35,36,37,38,39,40]. On the other hand, evidence regarding the impact of DMTs other than anti-CD20 agents or T cell responses on the incidence and severity of BIs is limited, as is the identification of clinical or immunological factors potentially associated with the time to swab reversion.

The identification of clinical and immune markers associated with reduced incidence or severity of SARS-CoV-2 infections and the timing of negativization are needed to improve the clinical management of PwMS under immunosuppressive therapies.

In this study, we found that 43.8% (28/64) of PwMS experienced a BI after three doses of COVID-19 mRNA vaccine. This relatively high incidence rate of SARS-CoV-2 infection is likely due to the spread of the Omicron variant in Italy, which coincided with the follow-up period of the study [41]. Omicron variants are known to be less pathogenic than the previous ones; however, they are highly transmissible despite vaccination [27,29,47]. The first-generation mRNA vaccines were designed against the spike protein of the Wuhan strain; thus, it is conceivable that they offered reduced protection against the Omicron variant that is highly mutated in the spike protein compared to any others [48,49].

The majority of BIs (75%) were mild, thus confirming the less pathogenic character of the Omicron variant and the protective role of vaccination against the severe disease. These results corroborate what was observed in larger cohorts of PwMS [40,50,51]. Only two cases of severe COVID-19 were reported in our cohort, specifically in two PwMS showing high disability levels. One patient was undergoing fingolimod therapy while the other one was treated with ocrelizumab.

Interestingly, the male population of our MS cohort seems to be more prone to BIs than females (61.9% males vs. 35% females). This greater susceptibility to COVID-19 may be ascribed to biological and genetic differences, particularly to the different innate and adaptive immune responses reported between males and females [52].

Among the clinical factors, the phenotype and severity of MS disease were associated with greater protection against infection. Having a relapsing-remitting phenotype significantly reduced the BI incidence rate in PwMS by 88% compared to those with a primary-progressive disease, which is in agreement with previous data [53]. Unlike the primary-progressive MS that progresses more gradually with the slow accumulation of neurological disability, the relapsing-remitting disease is characterized by relapses, which are exacerbations or attacks of neurological dysfunction, followed by remissions, which are periods without obvious disease activity [54].

Moreover, we found a significantly lower incidence rate of BIs in patients with greater MS severity (EDSS ≥ 3), although a higher EDSS, in those infected with SARS-CoV-2, was associated with an increased risk of severe COVID-19 [53,55,56]. Our results may be explained by the nature of the MS itself. MS is a central nervous system autoimmune disease in which damage to myelin causes symptoms including muscle weakness, thus resulting in walking difficulties and problems of coordination [54]. Therefore, it is reasonable to think that PwMS with a higher degree of disability have maintained greater social distancing measures, thus limiting the possibility of being infected. Consequently, this unexpected correlation between lower incidence of BIs and MS severity could be considered a reverse causality as the severity of the disease does not represent a protective factor for SARS-CoV-2 infection.

To note, SARS-CoV-2 infections in PwMS occurred after about 5 months from the third dose, a time corresponding to the initial waning of the humoral response as previously reported in several longitudinal studies [5,7,22,23,25,39]. A protective role of SARS-CoV-2 antibody levels against the Delta variant (HR = 0.57) and lower protection for the Omicron cases (HR = 1.40) was observed by Sormani and colleagues [35].

In this study, we observed a significantly lower seroconversion rate among subjects who had BI than uninfected subjects. Having an anti-RBD response ≥ 7.1 BAU/mL still conferred a 60% reduction of the BI incidence rate after adjusting for EDSS score and disease phenotype. Anti-RBD antibodies can be directed against different epitopes in the RBD of the spike protein, but not all of them can neutralize the infectious capacity of the virus [57]. Non-neutralizing antibodies are antibodies that can bind to viruses but cannot neutralize them. They can mediate protection against viruses through different strategies other than classical neutralization assays. For instance, non-neutralizing antibodies can mediate antibody-dependent cellular cytotoxicity, antibody-dependent cellular phagocytosis, and complement activation, and their binding can expose epitopes for neutralizing antibody binding possibly increasing the virus neutralization efficacy [58]. Our data highlight the important role of the antibody response in COVID-19 protection as supported by the higher frequency of BIs observed in ocrelizumab-treated patients (64.7%). Ocrelizumab is an anti-CD20 monoclonal antibody that acts by depleting B lymphocytes. This mechanism prevents PwMS treated with ocrelizumab from mounting an appropriate humoral response to vaccines or infections [5,6,7,33,37,39,59,60]. The lack of the antibody response makes ocrelizumab-treated patients a high-risk category for COVID-19 and longer persistence of SARS-CoV-2 infection, despite having a T cell-specific response [61].

Our results confirm what was reported by Novak and colleagues, who showed a rate of 59% in BIs after the third SARS-CoV-2 mRNA vaccination in ocrelizumab-treated patients without resulting in a severe outcome [33]. Moreover, it has been reported that patients treated with anti-CD20 agents showed a risk almost four times higher of having SARS-CoV-2 infection compared to patients under different DMTs with a hazard ratio (HR) of 3.72 [50].

As far as we know, this study showed for the first time an association between antibody and T cell-specific responses with a more rapid swab reversion. Specifically, having high anti-RBD antibody titers (≥ 809 BAU/mL) and an IFN-γ-specific T cell response significantly reduced the time required to obtain a negative swab by about 4.39 and 5.88 days, respectively, as confirmed by the multivariable analysis. On the contrary, the treatment with fingolimod significantly delayed the swab reversion by about 7 days. This result agrees with the mechanism of action of this immunosuppressive drug. Indeed, fingolimod is a sphingosine-1-phosphate receptor modulator that retains lymphocytes in the lymph nodes, thereby reducing the number of T and B cells in circulation [62]. Consequently, patients treated with fingolimod are incapable of mounting an appropriate T cell response to vaccines and infections [6,63].

Considering the pivotal role played by the cell-mediated immune response in fighting SARS-CoV-2 infections by removing infected cells [64], it is reasonable to think that the lack of T cell response in fingolimod-treated patients is responsible for the longer time taken for swab reversion.

Some limitations of the study need to be acknowledged. The small sample size of the cohort may have limited the emergence of significant associations. Moreover, the reduced number of cladribine-treated patients may have restricted their ability to demonstrate significant differences compared with other DMTs. However, the robustness of the reported data was confirmed by the multivariable analysis. The small number of PwMS with severe COVID-19 may have limited our ability to identify clinical and immunological factors associated with disease severity. The collected data were rigorously checked by the investigators and by those who carried out the analyses; however, we cannot completely rule out the presence of reporting bias. Furthermore, it is reasonable to think that precautions such as social distancing taken by patients with greater MS severity may have influenced the incidence of infection in our cohort.

On the other hand, this work has some strengths. This is a longitudinal and prospective study with a long-term follow-up. The cohort was well characterized both immunologically and clinically, allowing an in-depth analysis of SARS-CoV-2 incidence, severity, and timing of swab negativization. Moreover, we used standardized methods to detect antibody and T cell-specific responses.

## 5. Conclusions

In conclusion, this study provides evidence of the real-world impact of clinical and immunological data on both the incidence and severity of SARS-CoV-2 infection and on the timing of negativization in a cohort of PwMS undergoing DMTs after the third dose of COVID-19 mRNA vaccine. Our results showed that having a high antibody titer and T cell response favor a more rapid viral clearance, whereas treatment with fingolimod is associated with a delayed timing of swab reversion. These findings suggest different immunological protection from SARS-CoV-2 infection according to DMTs and, if confirmed in larger-scale population studies, they would be relevant to optimize the clinical management of PwMS.

## Figures and Tables

**Table 1:** Demographic and clinical characteristics of the 64 enrolled PwMS.

Patients’ Characteristics	
Total	64 (100)
**Age**, median (IQR) years	49 (41.5–55.5)
**Age class**, n (%)	
23–39	16 (25.0)
40–49	19 (29.7)
50–70	29 (45.3)
**Gender**: Female, n (%)	43 (67.2)
**Origin:** Italian, n (%)	62 (96.9)
**Presence of comorbidities**, n (%)	14 (21.9)
**BMI** (kg/m^2^), median (IQR)	24 (21–26)
**Vaccination-First dose with Comirnaty**, n (%)	59 (92.2)
**MS duration**, median (IQR) years	15 (7–25)
**MS treatment**, n (%)	
Cladribine	6 (9.4)
Fingolimod	26 (40.6)
Interferon beta	15 (23.4)
Ocrelizumab	17 (26.6)
**MS treatment duration**, median months (IQR)	5 (2–9)
**EDSS score**, median (IQR)	2 (0.5–4.3)
<3	37 (58.8)
≥3	27 (42.2)
**MS phenotype,** n (%)	
Primary-progressive (PP)	5 (7.8)
Relapsing-Remitting (RR)	59 (92.2)
**Lymphocytes count × 10^3^/µL**, median (IQR)	1.3 (0.7–1.7)
**Days from booster dose to sample**, median (IQR)	48 (43–51)
**Among PwMS with SARS-CoV-2 breakthrough infection**	28 (43.7)
**Days from 3rd dose to infection**, median (IQR)	155 (108–205)
**Days to negative swab**, median (IQR)	11 (9–15)
**COVID-19 therapy**, n (%)	
Anti-viral therapy	5 (17.9)
Monoclonal therapy	11 (39.3)
Non-steroidal anti-inflammatory drugs or paracetamol or no therapy *	12 (42.9)
**Severity of COVID-19**, n (%)	
Mild disease	21 (75.0)
Moderate disease	5 (17.9)
Severe disease	2 (7.1)

IQR: interquartile range; BMI: body mass index; EDSS: Expanded Disability Status Scale; PwMS: patients with multiple sclerosis; * two patients reported not having taken any therapy. In bold are reported the patient’s characteristics.

**Table 2:** Incidence of SARS-CoV-2 breakthrough infections according to the demographic and clinical characteristics of PwMS after the booster dose of COVID-19 vaccination.

Patients’ Characteristics	SARS-CoV-2 BIs			Univariable			Multivariable		
	No	Yes	Total	IRR	95%CI	*p*	IRR	95%CI	*p*
**Total**, n (%)	36 (56.3)	28 (43.8)	64 (100)						
**Age**, median (IQR) years	50 (45.5–58)	47.5 (38–53.5)	49 (41.5–55.5)						
23–39	8 (22.2)	8 (28.6)	16 (25.0)	Ref.					
40–49	10 (27.8)	9 (32.1)	19 (29.7)	1.01	0.39–2.63	0.979			
50–70	18 (50.0)	11 (39.3)	29 (45.3)	0.66	0.27–1.64	0.371			
**Gender**, n (%)									
Female	28 (77.8)	15 (53.6)	43 (67.2)	Ref.					
Male	8 (22.2)	13 (46.4)	21 (32.8)	2.19	1.04–4.6	**0.038**			
**Country of birth**, n (%)									
Italy	34 (94.4)	28 (100)	62 (96.9)	-	-	-			
Abroad	2 (5.6)	0 (0)	2 (3.1)	-	-	-			
**Presence of comorbidities**, n (%)									
No	27 (75.0)	23 (82.1)	50 (78.1)	Ref.					
Yes	9 (25.0)	5 (17.9)	14 (21.9)	0.69	0.26–1.83	0.460			
**BMI** (kg/m^2^), median (IQR)	24 (20.9–25.9)	24 (22–26.6)	24 (21.3–26.2)						
≤24	18 (50.0)	14 (50.0)	32 (50.0)	Ref.					
>24	18 (50.0)	14 (50.0)	32 (50.0)	1.03	0.49–2.16	0.938			
**Vaccination-First dose**, n (%)									
Comirnaty	33 (91.7)	26 (92.9)	59 (92.2)	Ref.					
Other	3 (8.3)	2 (7.1)	5 (7.8)	1.06	0.25–4.45	0.940			
**MS duration** (years), median (IQR)	16.2 (8.5–23.1)	12.9 (5.8–25)	15 (7.5–24.5)						
≤15	17 (47.2)	16 (57.1)	33 (51.6)	Ref.					
>15	19 (52.8)	12 (42.9)	31 (48.4)	0.79	0.37–1.67	0.539			
**MS treatment**, n (%)									
Cladribine	5 (13.9)	1 (3.6)	6 (9.4)	0.32	0.04–2.69	0.297			
Fingolimod	16 (44.4)	10 (35.7)	26 (40.6)	0.97	0.35–2.66	0.948			
Interferon beta	9 (25.0)	6 (21.4)	15 (23.4)	Ref.					
Ocrelizumab	6 (16.7)	11 (39.3)	17 (26.6)	2.21	0.82–5.97	0.118			
**MS treatment duration** (years), median (IQR)	6.5 (2–9.1)	3.7 (1.8–7.9)	5.1 (1.9–8.6)						
≤5	14 (38.9)	17 (60.7)	31 (48.4)	Ref.					
>5	22 (61.1)	11 (39.3)	33 (51.6)	0.49	0.23–1.04	0.062			
**EDSS score**, median (IQR)	3 (0.5–5)	1.8 (0.5–3)	2 (0.5–4.3)						
<3	17 (47.2)	20 (71.4)	37 (58.8)	Ref.			Ref.		
≥3	19 (52.8)	8 (28.6)	27 (42.2)	0.40	0.18–0.9	**0.029**	0.22	0.08–0.66	**0.006**
**MS phenotype**, n (%)									
Primary-progressive (PP)	1 (2.8)	4 (14.3)	5 (7.8)	Ref.			Ref.		
Relapsing-Remitting (RR)	35 (97.2)	24 (85.7)	59 (92.2)	0.33	0.11–0.94	**0.039**	0.12	0.03–0.46	**0.002**
**Lymphocytes count × 10^3^/µL,** median (IQR)	1.3 (0.7–1.6)	1.3 (0.7–1.8)	1.28 (0.65–1.67)						
≤1.28	18 (54.6)	11 (45.8)	29 (50.9)	Ref.					
>1.28	15 (45.4)	13 (54.2)	28 (49.1)	1.35	0.61–3.02	0.461			

BI: breakthrough infection; PwMS: patients with multiple sclerosis; IQR: interquartile range; BMI: body mass index; EDSS: Expanded Disability Status Scale; IRR: incidence rate ratio; CI: confidence interval; Ref: reference category; univariable Poisson regression. In bold are reported the patient’s characteristics and the significant values.

**Table 3:** Incidence rate ratios for SARS-CoV-2 infection in 64 PwMS according to antibody and T cell-specific immune response evaluated after 2–4 weeks from the booster dose.

Immune Response	No BI	BI	Total	Univariable			Multivariable		
	36 (56.3)	28 (43.8)	64 (100)	IRR	95%CI	*p*	aIRR	95%CI	*p*
**anti-RBD IgG** (BAU/mL)									
continuous, median (IQR)	352 (24–5513)	135 (1–1337)	272 (8–4222)						
score: Negative (<7.1)	5 (13.9)	11 (39.3)	16 (25.0)	Ref.			Ref.		
score: Positive (≥7.1)	31 (86.1)	17 (60.7)	48 (75.0)	0.33	0.15–0.7	**0.004**	0.40	0.17–0.97	**0.042**
**IFN-γ T cell-specific response** (pg/mL)									
continuous, median (IQR)	30 (1–222)	188 (1–578)	79 (1–349)						
<16	17 (47.2)	10 (35.7)	27 (42.2)	Ref.			Ref.		
≥16	19 (52.8)	18 (64.3)	37 (57.8)	1.61	0.74–3.49	0.228	0.55	0.22–1.34	0.187
**Neutralizing antibodies**									
continuous, median (IQR)	15 (5–320)	5 (5–160)	8 (5–320)						
<10	17 (47.2)	15 (53.6)	32 (50.0)	Ref.			Ref.		
≥10	19 (52.8)	13 (46.4)	32 (50.0)	0.83	0.39–1.74	0.618	0.68	0.30–1.55	0.362

PwMS: patients with multiple sclerosis; RBD: receptor-binding domain; IFN: interferon; BI: breakthrough infection; IRR: incidence rate ratio estimated with Poisson regression; aIRR: IRR adjusted for EDSS score and phenotype; CI: confidence interval; Ref: reference category. In bold are reported the patient’s immunologic variables and the significant values.

**Table 4:** Demographic and clinical characteristics of the 28 PwMS enrolled according to the COVID-19 severity.

Patients’ Characteristics	COVID-19 Severity				
	Mild Disease	Moderate Disease	Severe Disease	Total	*p*
**Total**	21 (75.0)	5 (17.9)	2 (7.1)	28 (100)	
**Age**, median (IQR) years	47 (39–53)	49 (30–55)	47.5 (46–49)	47.5 (38–53.5)	0.969
**Age class**, n (%)					0.511
23–39	6 (28.6)	2 (40)	0 (0)	8 (28.6)	
40–49	6 (28.6)	1 (20)	2 (100)	9 (32.1)	
50–70	9 (42.9)	2 (40)	0 (0)	11 (39.3)	
**Gender**, n (%)					0.293
Female	13 (61.9)	2 (40)	0 (0)	15 (53.6)	
Male	8 (38.1)	3 (60)	2 (100)	13 (46.4)	
**Presence of comorbidities**, n (%)					0.696
No	16 (76.2)	5 (100)	2 (100)	23 (82.1)	
Yes	5 (23.8)	0 (0)	0 (0)	5 (17.9)	
**BMI** (kg/m^2^), median (IQR)	24.2 (22.8–26.8)	22.1 (21.8–23.6)	24.5 (23.2–25.8)	24 (22–26.6)	0.571
**Vaccination-First dose type**, n (%)					1.000
Comirnaty	19 (90.5)	5 (100)	2 (100)	26 (92.9)	
Other	2 (9.5)	0 (0)	0 (0)	2 (7.1)	
**MS duration**, median (IQR) months(?)	15 (5.7–24.8)	10.7 (9.1–14)	19.1 (7.9–30.3)	12.9 (5.8–25)	0.763
**MS treatment**, n (%)					0.463
Cladribine	1 (4.8)	0 (0)	0 (0)	1 (3.6)	
Fingolimod	8 (38.1)	1 (20)	1 (50)	10 (35.7)	
Interferon beta	6 (28.6)	0 (0)	0 (0)	6 (21.4)	
Ocrelizumab	6 (28.6)	4 (80)	1 (50)	11 (39.3)	
**MS treatment duration**, median (IQR) months	4.7 (1.9–8)	1.9 (1.8–4.1)	6.7 (3.4–9.9)	3.7 (1.8–7.9)	0.310
**EDSS score**, median (IQR)	2 (0–2.5)	1.5 (1–1.5)	6.5 (6.5–6.5)	1.8 (0.5–3)	0.064
<3	16 (76.2)	4 (80.0)	0 (0)	20 (71.4)	0.089
≥3	5 (23.8)	1 (20.0)	2 (100)	8 (28.6)	
**MS phenotype,** n (%)					0.253
Primary-progressive (PP)	2 (9.5)	1 (20)	1 (50)	4 (14.3)	
Relapsing-Remitting (RR)	19 (90.5)	4 (80)	1 (50)	24 (85.7)	
**Lymphocytes count × 10^3^/µL,** median (IQR)	1.3 (0.7–1.7)	1.5 (1.2–1.7)	1.5 (0.2–2.7)	1.3 (0.7–1.8)	0.912
**COVID-19 therapy**, n(%)					0.098
Anti-viral therapy	4 (19.1)	1 (20.0)	0 (0)	5 (17.9)	
Monoclonal therapy	7 (33.3)	4 (80.0)	0 (0)	11 (39.3)	
NSAIDs, paracetamol or no therapy	10 (47.6)	0 (0)	2 (100)	12 (42.9)	
**Days from booster dose to infection**, median (IQR)	153 (105–169)	204 (154–204)	263 (258–268)	154.5 (108–204.5)	0.129
**anti-RBD IgG** (BAU/mL), median (IQR)					
continuous	407 (2–2319)	1 (0–4)	268 (0–537)	135 (1–1337)	0.160
<7.1, n (%)	6 (28.6)	4 (80)	1 (50)	11 (39.3)	0.090
≥7.1, n (%)	15 (71.4)	1 (20)	1 (50)	17 (60.7)	
**IFN-γ T cell-specific response** (pg/mL), median (IQR)					
continuous	166 (1–564)	238 (207–353)	365 (0–729)	188 (1–578)	0.957
<16, n (%)	8 (38.1)	1 (20)	1 (50)	10 (35.7)	0.823
≥16, n (%)	13 (61.9)	4 (80)	1 (50)	18 (64.3)	
**Neutralizing antibodies**, median (IQR)					
continuous	20 (5–160)	5 (5–5)	23 (5–40)	5 (5–160)	0.323
<10, n (%)	10 (47.6)	4 (80)	1 (50)	15 (53.6)	0.655
≥10, n (%)	11 (52.4)	1 (20)	1 (50)	13 (46.4)	

IQR: interquartile range; BMI: body mass index; NSAIDs, non-steroidal anti-inflammatory drugs; PwMS: patients with multiple sclerosis; RBD: receptor-binding domain; IFN: interferon; EDSS: Expanded Disability Status Scale. In bold are reported the patient’s characteristics.

**Table 5:** Days to SARS-CoV-2 clearance in 25 PwMS with breakthrough infections.

	Quantile Regression Model					
Patient’s Characteristic	Univariable			Multivariable		
	Coefficient *	95%CI	*p*	Coefficient **	95%CI	*p*
**Age class**, years n (%)						
23–39	Ref.					
40–49	3.00	−3.07; 9.07	0.317			
50–70	0.00	−5.78; 5.78	1.000			
**Gender**, n (%)						
Female	Ref.					
Male	0.00	−5.03; 5.03	1.000			
**Presence of comorbidities**, n (%)						
No	Ref.					
Yes	−5.00	−10.6; 0.6	0.078			
**BMI** (kg/m^2^), median (IQR)	−0.21	−0.79; 0.37	0.459			
**Vaccination-First dose**, n (%)						
Comirnaty	Ref.					
Other	−3.00	−11.63; 5.63	0.479			
**MS duration**, median (IQR) years	0.00	−0.29; 0.29	1.000			
**MS treatment**, n (%)						
Cladribine	0.00	−8.52; 8.52	1.000			
Fingolimod	**7.00**	**2.84; 11.16**	**0.002**			
Interferon beta	Ref.					
Ocrelizumab	3.00	−1.16; 7.16	0.148			
**MS treatment duration**, median (IQR) years	−0.41	−0.76; −0.05	0.028			
**EDSS score**, median (IQR)	0.00	−1.32; 1.32	1.000			
<3	Ref.					
≥3	0.00	−5.13; 5.13	1.000			
**MS phenotype**, n (%)						
Primary-progressive (PP)	Ref.					
Relapsing-Remitting (RR)	0.00	−7.2; 7.2	1.000			
**Lymphocytes count × 10^3^/µL,** median (IQR)	−2.36	−6.46; 1.75	0.244			
**COVID-19 therapy**, n (%)						
Anti-viral therapy	Ref.					
Monoclonal therapy	4.00	−2.3; 10.34	0.204			
NSAIDs, paracetamol or no therapy	−1.00	−7.13; 6.13	0.738			
**Severity of COVID-19**, n (%)						
Mild illness	Ref.					
Moderate illness	4	−1.90; 9.90	0.174			
Severe illness	1	−11.0; 13.0	0.865			
**Days from booster dose to sample**, median (IQR)	0.04	−0.3; 0.39	0.796			
**Anti-RBD IgG** (BAU/mL), median (IQR)						
continuous	−0.0004	−0.001; 0.0003	0.245	−0.001	−0.001; 0.0002	0.163
score: Negative (<7.1), n (%)	Ref.			Ref.		
score: Positive (≥7.1), n (%)	−4.00	−8.31; 0.31	0.067	−2.73	−7.27; 1.81	0.225
<809	Ref.			Ref.		
≥809	**−6.00**	**−10.7; −1.3**	**0.015**	**−4.39**	**−8.8; 0.01**	**0.051**
**IFN-γ T cell-specific response** (pg/mL), median (IQR)						
continuous	−0.0018	−0.006; 0.003	0.422	−0.002	−0.006; 0.003	0.446
<16, n (%)	Ref.			Ref.		
≥16, n (%)	**−5.00**	**−9.77; −0.23**	**0.041**	**−5.88**	**−10.65; −1.10**	**0.018**
**Neutralizing antibodies**, median (IQR)						
continuous	−0.01	−0.03; 0.01	0.178	−0.01	−0.03; 0	0.135
<10, n (%)	Ref.			Ref.		
≥10, n (%)	0.00	−3.71; 3.71	1.000	−2.5	−7.5; 2.5	0.310

CI: confidence interval; Ref: reference category; NSAIDs, non-steroidal anti-inflammatory drugs; PwMS: patients with multiple sclerosis; RBD: receptor-binding domain; IFN: interferon; EDSS: Expanded Disability Status Scale. * Estimated by univariable median regression model; ** estimated by median regression model after adjusting for the time elapsed from sample collection. In bold are reported the patient’s characteristics and the significant values.

## Data Availability

The original contributions presented in the study are included in the article/Supplementary Material, and further inquiries can be directed to the corresponding author/s.

## Supplementary Materials

The following are available online at https://www.mdpi.com/article/10.3390/vaccines12080926/s1, Figure S1: IFN-γ-specific T cell response after stimulation with staphylococcal enterotoxin B (SEB), Table S1: COVID-19 therapy.

## References

[1] Coronavirus Disease (COVID-19)—World Health Organization. https://www.who.int/emergencies/diseases/novel-coronavirus-2019.

[2] Grifoni A., Alonzi T., Alter G., Noonan D.M., Landay A.L., Albini A., Goletti D. (2023). Impact of Aging on Immunity in the Context of COVID-19, HIV, and Tuberculosis. Front. Immunol..

[3] Li Y., Choudhary M.C., Regan J., Boucau J., Nathan A., Speidel T., Liew M.Y., Edelstein G.E., Kawano Y., Uddin R. (2024). SARS-CoV-2 Viral Clearance and Evolution Varies by Type and Severity of Immunodeficiency. Sci. Transl. Med..

[4] Petrone L., Tortorella C., Aiello A., Farroni C., Ruggieri S., Castilletti C., Meschi S., Cuzzi G., Vanini V., Palmieri F. (2022). Humoral and Cellular Response to Spike of Delta SARS-CoV-2 Variant in Vaccinated Patients with Multiple Sclerosis. Front. Neurol..

[5] Ruggieri S., Aiello A., Tortorella C., Navarra A., Vanini V., Meschi S., Lapa D., Haggiag S., Prosperini L., Cuzzi G. (2023). Dynamic Evolution of Humoral and T-Cell Specific Immune Response to COVID-19 mRNA Vaccine in Patients with Multiple Sclerosis Followed until the Booster Dose. Int. J. Mol. Sci..

[6] Tortorella C., Aiello A., Gasperini C., Agrati C., Castilletti C., Ruggieri S., Meschi S., Matusali G., Colavita F., Farroni C. (2022). Humoral- and T-Cell-Specific Immune Responses to SARS-CoV-2 mRNA Vaccination in Patients with MS Using Different Disease-Modifying Therapies. Neurology.

[7] Aiello A., Coppola A., Ruggieri S., Farroni C., Altera A.M.G., Salmi A., Vanini V., Cuzzi G., Petrone L., Meschi S. (2023). Longitudinal Characterisation of B and T-Cell Immune Responses after the Booster Dose of COVID-19 mRNA-Vaccine in People with Multiple Sclerosis Using Different Disease-Modifying Therapies. J. Neurol. Neurosurg. Psychiatry.

[8] Castelo-Branco A., Chiesa F., Conte S., Bengtsson C., Lee S., Minton N., Niemcryk S., Lindholm A., Rosenlund M., Piehl F. (2020). Infections in Patients with Multiple Sclerosis: A National Cohort Study in Sweden. Mult. Scler. Relat. Disord..

[9] McGinley M.P., Goldschmidt C.H., Rae-Grant A.D. (2021). Diagnosis and Treatment of Multiple Sclerosis: A Review. JAMA.

[10] Iyer R.B., Raghavendra S., Nooraine J., Jaychandran R. (2022). COVID-19 Outcomes in Persons with Multiple Sclerosis Treated with Rituximab. Mult. Scler. Relat. Disord..

[11] Foerch C., Friedauer L., Bauer B., Wolf T., Adam E.H. (2020). Severe COVID-19 Infection in a Patient with Multiple Sclerosis Treated with Fingolimod. Mult. Scler. Relat. Disord..

[12] Simpson-Yap S., De Brouwer E., Kalincik T., Rijke N., Hillert J.A., Walton C., Edan G., Moreau Y., Spelman T., Geys L. (2021). Associations of Disease-Modifying Therapies With COVID-19 Severity in Multiple Sclerosis. Neurology.

[13] Zaloum S.A., Wood C.H., Tank P., Upcott M., Vickaryous N., Anderson V., Baker D., Chance R., Evangelou N., George K. (2023). Risk of COVID-19 in People with Multiple Sclerosis Who Are Seronegative Following Vaccination. Mult. Scler. J..

[14] Prosperini L., Tortorella C., Haggiag S., Ruggieri S., Galgani S., Gasperini C. (2022). Increased Risk of Death from COVID-19 in Multiple Sclerosis: A Pooled Analysis of Observational Studies. J. Neurol..

[15] Achiron A., Mandel M., Dreyer-Alster S., Harari G., Magalashvili D., Sonis P., Dolev M., Menascu S., Flechter S., Falb R. (2021). Humoral Immune Response to COVID-19 mRNA Vaccine in Patients with Multiple Sclerosis Treated with High-Efficacy Disease-Modifying Therapies. Ther. Adv. Neurol. Disord..

[16] Sabatino J.J., Mittl K., Rowles W.M., McPolin K., Rajan J.V., Laurie M.T., Zamecnik C.R., Dandekar R., Alvarenga B.D., Loudermilk R.P. (2022). Multiple Sclerosis Therapies Differentially Affect SARS-CoV-2 Vaccine–Induced Antibody and T Cell Immunity and Function. JCI Insight.

[17] Sainz de la Maza S., Walo-Delgado P.E., Rodríguez-Domínguez M., Monreal E., Rodero-Romero A., Chico-García J.L., Pariente R., Rodríguez-Jorge F., Ballester-González R., Villarrubia N. (2023). Short- and Long-Term Humoral and Cellular Immune Responses to SARS-CoV-2 Vaccination in Patients with Multiple Sclerosis Treated with Disease-Modifying Therapies. Vaccines.

[18] Apostolidis S.A., Kakara M., Painter M.M., Goel R.R., Mathew D., Lenzi K., Rezk A., Patterson K.R., Espinoza D.A., Kadri J.C. (2021). Cellular and Humoral Immune Responses Following SARS-CoV-2 mRNA Vaccination in Patients with Multiple Sclerosis on Anti-CD20 Therapy. Nat. Med..

[19] Habek M., Željko C., Savić Mlakar A., Bendelja K., Rogić D., Adamec I., Barun B., Gabelić T., Krbot Skorić M. (2022). Humoral and Cellular Immunity in Convalescent and Vaccinated COVID-19 People with Multiple Sclerosis: Effects of Disease Modifying Therapies. Mult. Scler. Relat. Disord..

[20] Meyer-Arndt L., Braun J., Fauchere F., Vanshylla K., Loyal L., Henze L., Kruse B., Dingeldey M., Jürchott K., Mangold M. (2022). SARS-CoV-2 mRNA Vaccinations Fail to Elicit Humoral and Cellular Immune Responses in Patients with Multiple Sclerosis Receiving Fingolimod. J. Neurol. Neurosurg. Psychiatry.

[21] Shrotri M., Navaratnam A.M.D., Nguyen V., Byrne T., Geismar C., Fragaszy E., Beale S., Fong W.L.E., Patel P., Kovar J. (2021). Spike-Antibody Waning after Second Dose of BNT162b2 or ChAdOx1. Lancet.

[22] Bajwa H.M., Novak F., Nilsson A.C., Nielsen C., Holm D.K., Østergaard K., Witt A.H., Byg K.-E., Johansen I.S., Mittl K. (2022). Persistently Reduced Humoral and Sustained Cellular Immune Response from First to Third SARS-CoV-2 mRNA Vaccination in Anti-CD20-Treated Multiple Sclerosis Patients. Mult. Scler. Relat. Disord..

[23] Levin E.G., Lustig Y., Cohen C., Fluss R., Indenbaum V., Amit S., Doolman R., Asraf K., Mendelson E., Ziv A. (2021). Waning Immune Humoral Response to BNT162b2 Covid-19 Vaccine over 6 Months. N. Engl. J. Med..

[24] Gillot C., Bayart J.-L., Closset M., Cabo J., Maloteau V., Dogné J.-M., Douxfils J., Favresse J. (2023). Peri-Infection Titers of Neutralizing and Binding Antibodies as a Predictor of COVID-19 Breakthrough Infections in Vaccinated Healthcare Professionals: Importance of the Timing. Clin. Chem. Lab. Med..

[25] Menegale F., Manica M., Zardini A., Guzzetta G., Marziano V., d’Andrea V., Trentini F., Ajelli M., Poletti P., Merler S. (2023). Evaluation of Waning of SARS-CoV-2 Vaccine-Induced Immunity: A Systematic Review and Meta-Analysis. JAMA Netw. Open.

[26] Machkovech H.M., Hahn A.M., Garonzik Wang J., Grubaugh N.D., Halfmann P.J., Johnson M.C., Lemieux J.E., O’Connor D.H., Piantadosi A., Wei W. (2024). Persistent SARS-CoV-2 Infection: Significance and Implications. Lancet Infect. Dis..

[27] Accorsi E.K., Britton A., Fleming-Dutra K.E., Smith Z.R., Shang N., Derado G., Miller J., Schrag S.J., Verani J.R. (2022). Association Between 3 Doses of mRNA COVID-19 Vaccine and Symptomatic Infection Caused by the SARS-CoV-2 Omicron and Delta Variants. JAMA.

[28] Ioannou G.N., Bohnert A.S.B., O’Hare A.M., Boyko E.J., Maciejewski M.L., Smith V.A., Bowling C.B., Viglianti E., Iwashyna T.J., Hynes D.M. (2022). Effectiveness of mRNA COVID-19 Vaccine Boosters Against Infection, Hospitalization, and Death: A Target Trial Emulation in the Omicron (B.1.1.529) Variant Era. Ann. Intern. Med..

[29] Andrews N., Stowe J., Kirsebom F., Toffa S., Rickeard T., Gallagher E., Gower C., Kall M., Groves N., O’Connell A.-M. (2022). Covid-19 Vaccine Effectiveness against the Omicron (B.1.1.529) Variant. N. Engl. J. Med..

[30] Tsang N.N.Y., So H.C., Cowling B.J., Leung G.M., Ip D.K.M. (2023). Effectiveness of BNT162b2 and CoronaVac COVID-19 Vaccination against Asymptomatic and Symptomatic Infection of SARS-CoV-2 Omicron BA.2 in Hong Kong: A Prospective Cohort Study. Lancet Infect. Dis..

[31] Nyberg T., Ferguson N.M., Nash S.G., Webster H.H., Flaxman S., Andrews N., Hinsley W., Bernal J.L., Kall M., Bhatt S. (2022). Comparative Analysis of the Risks of Hospitalisation and Death Associated with SARS-CoV-2 Omicron (B.1.1.529) and Delta (B.1.617.2) Variants in England: A Cohort Study. Lancet.

[32] Calabrese C.M., Kirchner E., Husni E.M., Moss B.P., Fernandez A.P., Jin Y., Calabrese L.H. (2022). Breakthrough SARS-CoV-2 Infections in Patients with Immune-Mediated Disease Undergoing B Cell-Depleting Therapy: A Retrospective Cohort Analysis. Arthritis Rheumatol..

[33] Novak F., Bajwa H.M., Coia J.E., Nilsson A.C., Nielsen C., Holm D.K., Østergaard K., Hvidt M.V.M., Byg K.-E., Johansen I.S. (2023). Low Protection from Breakthrough SARS-CoV-2 Infection and Mild Disease Course in Ocrelizumab-Treated Patients with Multiple Sclerosis after Three mRNA Vaccine Doses. J. Neurol. Neurosurg. Psychiatry.

[34] Cross A.H., Delgado S., Habek M., Davydovskaya M., Ward B.J., Cree B.A.C., Totolyan N., Pingili R., Mancione L., Hu X. (2022). Correction to: COVID-19 Outcomes and Vaccination in People with Relapsing Multiple Sclerosis Treated with Ofatumumab. Neurol. Ther..

[35] Sormani M.P., Schiavetti I., Inglese M., Carmisciano L., Laroni A., Lapucci C., Visconti V., Serrati C., Gandoglia I., Tassinari T. (2022). Breakthrough SARS-CoV-2 Infections after COVID-19 mRNA Vaccination in MS Patients on Disease Modifying Therapies during the Delta and the Omicron Waves in Italy. EBioMedicine.

[36] Disanto G., Galante A., Cantu’ M., Sacco R., Mele F., Eisler J.J., Keller F., Bernasconi E., Sallusto F., Zecca C. (2023). Longitudinal Postvaccine SARS-CoV-2 Immunoglobulin G Titers, Memory B-Cell Responses, and Risk of COVID-19 in Multiple Sclerosis Over 1 Year. Neurol. Neuroimmunol. Neuroinflamm..

[37] Holroyd K.B., Healy B.C., Conway S., Houtchens M., Bakshi R., Bhattacharyya S., Bose G., Galetta K., Kaplan T., Severson C. (2022). Humoral Response to COVID-19 Vaccination in MS Patients on Disease Modifying Therapy: Immune Profiles and Clinical Outcomes. Mult. Scler. Relat. Disord..

[38] Klineova S., Farber R.S., DeAngelis T., Leung T., Smith T., Blanck R., Zhovtis-Ryerson L., Harel A. (2023). Vaccine-Breakthrough SARS-CoV-2 Infections in People with Multiple Sclerosis and Related Conditions: An Observational Study by the New York COVID-19 Neuro-Immunology Consortium (NYCNIC-2). Mult. Scler. J..

[39] Jakimovski D., Zakalik K., Awan S., Kavak K.S., Pennington P., Hojnacki D., Kolb C., Lizarraga A.A., Eckert S.P., Sarrosa R. (2022). COVID-19 Vaccination in Multiple Sclerosis and Inflammatory Diseases: Effects from Disease-Modifying Therapy, Long-Term Seroprevalence and Breakthrough Infections. Vaccines.

[40] van Kempen Z.L.E., Stalman E.W., Steenhuis M., Kummer L.Y.L., van Dam K.P.J., Wilbrink M.F., Ten Brinke A., van Ham S.M., Kuijpers T., Rispens T. (2023). SARS-CoV-2 Omicron Breakthrough Infections in Patients with Multiple Sclerosis. J. Neurol. Neurosurg. Psychiatry.

[41] Stefanelli P., Trentini F., Petrone D., Mammone A., Ambrosio L., Manica M., Guzzetta G., d’Andrea V., Marziano V., Zardini A. (2022). Tracking the Progressive Spread of the SARS-CoV-2 Omicron Variant in Italy, December 2021 to January 2022. Eurosurveillance.

[42] Thompson A.J., Banwell B.L., Barkhof F., Carroll W.M., Coetzee T., Comi G., Correale J., Fazekas F., Filippi M., Freedman M.S. (2018). Diagnosis of Multiple Sclerosis: 2017 Revisions of the McDonald Criteria. Lancet Neurol..

[43] Clinical Spectrum. https://www.covid19treatmentguidelines.nih.gov/overview/clinical-spectrum/.

[44] Aiello A., Najafi Fard S., Petruccioli E., Petrone L., Vanini V., Farroni C., Cuzzi G., Navarra A., Gualano G., Mosti S. (2021). Spike Is the Most Recognized Antigen in the Whole-Blood Platform in Both Acute and Convalescent COVID-19 Patients. Int. J. Infect. Dis..

[45] Matusali G., Colavita F., Lapa D., Meschi S., Bordi L., Piselli P., Gagliardini R., Corpolongo A., Nicastri E., Antinori A. (2021). SARS-CoV-2 Serum Neutralization Assay: A Traditional Tool for a Brand-New Virus. Viruses.

[46] Faul F., Erdfelder E., Buchner A., Lang A.-G. (2009). Statistical Power Analyses Using G*Power 3.1: Tests for Correlation and Regression Analyses. Behav. Res. Methods.

[47] Suzuki R., Yamasoba D., Kimura I., Wang L., Kishimoto M., Ito J., Morioka Y., Nao N., Nasser H., Uriu K. (2022). Attenuated Fusogenicity and Pathogenicity of SARS-CoV-2 Omicron Variant. Nature.

[48] Cao Y., Wang J., Jian F., Xiao T., Song W., Yisimayi A., Huang W., Li Q., Wang P., An R. (2022). Omicron Escapes the Majority of Existing SARS-CoV-2 Neutralizing Antibodies. Nature.

[49] Liu L., Iketani S., Guo Y., Chan J.F.-W., Wang M., Liu L., Luo Y., Chu H., Huang Y., Nair M.S. (2022). Striking Antibody Evasion Manifested by the Omicron Variant of SARS-CoV-2. Nature.

[50] Immovilli P., Schiavetti I., Franceschini A., De Mitri P., Gelati L., Rota E., Guidetti D. (2024). Breakthrough COVID-19 in People with Multiple Sclerosis on Disease Modifying Treatments: Is It Still a Severe Disease?. Mult. Scler. Relat. Disord..

[51] Spierer R., Lavi I., Bloch S., Mazar M., Golan D. (2023). Risk of Breakthrough COVID-19 after Vaccination among People with Multiple Sclerosis on Disease-Modifying Therapies. J. Neurol..

[52] Plebani M., Lippi G. (2022). Sex and gender differences in COVID-19: A narrative review. J. Sex Gend. Specif. Med..

[53] Chaudhry F., Bulka H., Rathnam A.S., Said O.M., Lin J., Lorigan H., Bernitsas E., Rube J., Korzeniewski S.J., Memon A.B. (2020). COVID-19 in Multiple Sclerosis Patients and Risk Factors for Severe Infection. J. Neurol. Sci..

[54] Dobson R., Giovannoni G. (2019). Multiple Sclerosis—A Review. Eur. J. Neurol..

[55] Louapre C., Collongues N., Stankoff B., Giannesini C., Papeix C., Bensa C., Deschamps R., Créange A., Wahab A., Pelletier J. (2020). Clinical Characteristics and Outcomes in Patients with Coronavirus Disease 2019 and Multiple Sclerosis. JAMA Neurol..

[56] Immovilli P., Schiavetti I., Cordioli C., De Mitri P., Grazioli S., Guidetti D., Sormani M.P. (2022). Lung Involvement Correlates with Disability in MS Patients with COVID-19 Pneumonia. Neurol. Sci..

[57] Yuan M., Liu H., Wu N.C., Lee C.-C.D., Zhu X., Zhao F., Huang D., Yu W., Hua Y., Tien H. (2020). Structural Basis of a Shared Antibody Response to SARS-CoV-2. Science.

[58] Chandler T.L., Yang A., Otero C.E., Permar S.R., Caddy S.L. (2023). Protective Mechanisms of Nonneutralizing Antiviral Antibodies. PLoS Pathog..

[59] Katz J.D., Bouley A.J., Jungquist R.M., Douglas E.A., O’Shea I.L., Lathi E.S. (2022). Humoral and T-Cell Responses to SARS-CoV-2 Vaccination in Multiple Sclerosis Patients Treated with Ocrelizumab. Mult. Scler. Relat. Disord..

[60] Räuber S., Korsen M., Huntemann N., Rolfes L., Müntefering T., Dobelmann V., Hermann A.M., Kölsche T., Lipinski K., von Wnuck Lipinski K. (2022). Immune Response to SARS-CoV-2 Vaccination in Relation to Peripheral Immune Cell Profiles among Patients with Multiple Sclerosis Receiving Ocrelizumab. J. Neurol. Neurosurg. Psychiatry.

[61] Januel E., Hajage D., Labauge P., Maillart E., De Sèze J., Zephir H., Pelletier J., Guilloton L., Bensa C., Heinzlef O. (2023). Association Between Anti-CD20 Therapies and COVID-19 Severity Among Patients with Relapsing-Remitting and Progressive Multiple Sclerosis. JAMA Netw. Open.

[62] Chun J., Hartung H.-P. (2010). Mechanism of Action of Oral Fingolimod (FTY720) in Multiple Sclerosis. Clin. Neuropharmacol..

[63] Palomares Cabeza V., Kummer L.Y.L., Wieske L., Hagen R.R., Duurland M., Konijn V.A.L., van Dam K.P.J., Stalman E.W., van de Sandt C.E., Boekel L. (2022). Longitudinal T-Cell Responses after a Third SARS-CoV-2 Vaccination in Patients with Multiple Sclerosis on Ocrelizumab or Fingolimod. Neurol. Neuroimmunol. Neuroinflamm..

[64] Petrone L., Sette A., de Vries R.D., Goletti D. (2023). The Importance of Measuring SARS-CoV-2-Specific T-Cell Responses in an Ongoing Pandemic. Pathogens.

