# Supplementary material for: Anti-RBD Antibody Levels and IFN-γ-Specific T Cell Response Are Associated with a More Rapid Swab Reversion in Patients with Multiple Sclerosis after the Booster Dose of COVID-19 Vaccination
Source: Vaccines (Basel). 2024 Aug 19;12(8):926. doi: 10.3390/vaccines12080926 (PMC11359508; doi:10.3390/vaccines12080926)
Supplement: Supplementary file 1 [file vaccines-12-00926-s001.zip › Supplementary figure.pdf]

Supplementary Figure

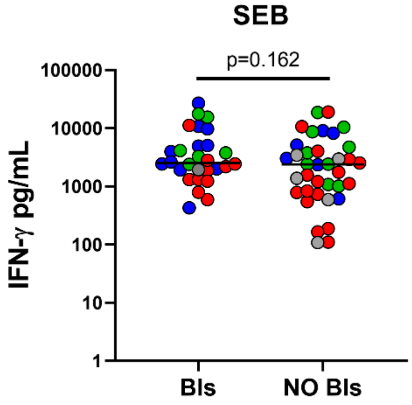

SEB responders N (%)	28/28 (100%)	36/36 (100%)	p>0.999

Supplementary Figure S1. IFN-γ-specific T-cell response after stimulation with staphylococcal enterotoxin B (SEB). The enrolled PwMS (n=64) were stratified into two groups: BIs group (n=28), which included those who had COVID-19 after the third dose, and no BIs group (n=36), which included those who did not have COVID-19 during the follow-up period. IFN-γ levels were subtracted from the unstimulated-control value and reported in pg/mL. Each colour dot represents a different treatment as shown in the legend. Mann-Whitney test was performed for pairwise comparison and Fisher Exact test was to compare the proportion of responders. A p-value < 0.05 was considered significant. Abbreviations: COVID-19, COronaVIrus Disease 2019; PwMS, patients with multiple sclerosis; BIs, breakthrough infections; IFN, interferon; N, number.
