# Supplementary material for: Anti-RBD Antibody Levels and IFN-γ-Specific T Cell Response Are Associated with a More Rapid Swab Reversion in Patients with Multiple Sclerosis after the Booster Dose of COVID-19 Vaccination
Source: Vaccines (Basel). 2024 Aug 19;12(8):926. doi: 10.3390/vaccines12080926 (PMC11359508; doi:10.3390/vaccines12080926)
Supplement: Supplementary file 1 [file vaccines-12-00926-s001.zip › Supplementary table.pdf]

Supplementary Table S1

MS treatment	COVID-19 therapy			
	Antiviral N (%)	Monoclonal N (%)	NSAIDs, paracetamol or no therapy N (%)	Total N (%)
**Cladribine**	1 (100)	0 (0)	0 (0)	1 (100)
**Fingolimod**	1 (11.1)	5 (55.6)	3 (33.3)	9 (100)
**Interferon beta**	0 (0)	0 (0)	6 (100)	6 (100)
**Ocrelizumab**	3 (33.3)	4 (44.4)	2 (22.2)	9 (100)
**Total N (%)**	5 (20)	9 (36)	11 (44)	25 (100)

Footnotes: COVID-19, COronaVIrus Disease 2019; NSAIDs, Non-steroidal anti-inflammatory drugs; MS: multiple sclerosis; N, number.
